# What drivers will influence global immunizations in the era of grand convergence in global health?

**DOI:** 10.1016/j.vaccine.2016.09.071

**Published:** 2017-01-20

**Authors:** Orin S. Levine

**Affiliations:** Vaccine Delivery Program, Global Development Division, Bill & Melinda Gates Foundation, 500 Fifth Avenue North, Seattle, WA 98109, United States

**Keywords:** Vaccines, Immunization, Policy, Grand convergence, Developing countries, Vaccine delivery, Epidemics

## Abstract

Recent projections suggest that by 2035 global health will look dramatically different than it does today. In what’s called a ‘grand convergence’ the world is likely to be characterized by far more similarities than differences in the prevailing health and medical problems across populations. This manuscript considers how key drivers for vaccine use and uptake might change as a result of the grand convergence and how decisions taken now might anticipate those changes in ways that position immunizations to continue playing an important role in the future.

## 1 Introduction

Recent projections from an international consortium of health scientists and economists forecast that with a concerted effort to scale up existing interventions and develop new ones we could see a ‘grand convergence’ in public health (GCIPH) by 2035 [1]. In essence, this report projects a massive narrowing in health disparities between low and middle income countries (LMIC) and rich countries focused on a few key indicators of population health such as under 5 mortality rates and the incidence of new HIV or TB infections by the year 2035. With this GCIPH, it is projected that the predominant pattern of population health in LMIC will be what is now typically seen in ‘middle income’ countries - a much lower burden of communicable diseases and a more substantial portion of the total disease burden being accounted for by non-communicable diseases.

This grand convergence in global health is far from a foregone conclusion but the trends in place today suggest that, like the Millennium Development Goals, these levels of health are likely to be met in at least some, maybe a large number of countries. With this potential change, and since successfully developing new vaccines requires upwards of 10–15 years, this paper considers how different drivers might influence vaccine development and introduction by the year 2035 and speculates on some of the implications for vaccine decision making of those drivers.

I hypothesize that 6 key drivers will increasingly influence the demand for and use of vaccines in the era of 2035 and beyond. None of these drivers is new. Each already contributes to vaccine development and implementation decisions today. However, I propose that by 2035 the relative influence of these drivers will be more substantial than it is today.

### 1.1 Epidemic potential

In an increasingly interconnected world, where international travel allows diseases to cross borders with alacrity, we should expect added attention to the issue of prevention of diseases of epidemic potential, and subsequently, increased prioritization for vaccines that can prevent such epidemics. Recent outbreaks of Zika virus and Ebola virus disease are clear evidence of the health and economic disruption that results from epidemic diseases that occur in regions or sub-regions. Additional efforts to improve the global community’s ability to respond to epidemics by strengthening surveillance and outbreak response are in development to facilitate a more effective response when new threats appear. However, a coordinated sustainable proactive program to develop and make available vaccines and other immunologics that diminish the likelihood of an outbreak taking hold should also be expected.

### 1.2 Localized epidemiologic need

The first set of globally recommended vaccines were largely vaccines that either were transmitted person-to-person (e.g., polio, measles, pertussis, diphtheria, tuberculosis) or existed everywhere in an environmental reservoir like the soil (e.g., tetanus). In other words, advanced levels of health infrastructure and environmental development did not eliminate the risk of the disease. This focus on globally relevant person-to-person infections extended into the next round of global vaccines against diseases like hepatitis, meningitis, and cervical cancer. Many of these global infections are now vaccine preventable and it is likely that diseases that have wide variations in local risk will gain increased attention for vaccine development and use.

Vector-borne infections are an obvious example, where the vector doesn’t exist the disease is not going to be transmitted, and there is little demand for the vaccine but where the disease exists its often a top local priority. Malaria and dengue are obvious examples. While markets for travelers may also be important for these vaccines, the locales where the diseases are prevalent will be the main drivers for the development and use of these vaccines. In the case of dengue, the *Aedes aegypti* mosquito that transmits the virus is distributed across the tropics, the burden of dengue disease appears to be concentrated in Asia and Latin America and not to be prevalent in Africa [2], [3]. That pattern may change over time but for now it demonstrates that even within the range of a vector the rationale for use of a vaccine may be geographically focal.

Interestingly even within person to person transmitted diseases like meningococcal disease we see examples of diverging patterns of local epidemiology driving to locally oriented vaccine solutions. Consider the case of meningococcal disease where serogroup A vaccines have been developed and manufactured for the meningitis belt of Africa [4] but in industrialized countries the most recent efforts were focused on developing safe, effective serogroup B vaccines [5]. In this way each vaccine is suited to the local epidemiology but no one vaccine is made to suit all geographies.

### 1.3 Vaccine safety

As the incidence of vaccine preventable diseases declines, presumably due to control of the disease by vaccination but also influenced by other environmental and host factors, it has been observed that communities will begin to focus more on the adverse effects associated with vaccination than with the adverse effects of the disease itself [6]. This paradoxical interaction would foreshadow a predictable increase in vaccine safety concerns as disease rates decrease, and potentially an increase in vaccine hesitancy, as a consequence in the years ahead.

In current developing country vaccine programs, vaccine safety is an important consideration and every effort is made to deliver immunizations as safely as possible. This effort has, to date, focused on issues associated with the administration of the vaccine with innovations such as the vaccine vial monitor and the auto-disable syringe as examples of ways technology has helped to make immunization safer in developing country environments [7], [8]. However, in making the decisions to procure vaccines, vaccine safety is just one of the product characteristics considered and in many cases, other characteristics of a vaccine, besides the frequency of adverse events, take precedent in selecting the vaccine. For example, in developing country programs the superior efficacy, duration of protection, and lower priced but more reactogenic whole-cell pertussis combination vaccines are typically preferred over the less reactogenic but higher priced, less efficacious acellular pertussis vaccines. Similarly, in countries using mumps vaccine, vaccines based upon the more reactogenic Urabe strain are often used. In wealthier countries this is typically reversed and may portend a future where countries will increasingly prioritize, and pay for a less reactogenic vaccine when the perceived threat of disease decreases.

### 1.4 Delivery system strength

From 1974 to 2010 the global expanded program on immunization in developing countries delivered just a few vaccines and depended solely on a handful of contact points. Each contact point required administration of typically one, or at most, two injectable vaccines and one oral vaccine. With the success of vaccine development and spurred in part by Gavi, the Vaccine Alliance, this situation is going to be vastly different in 2020 and beyond. Some countries emerging into middle-income status such as Ghana now deliver a far wider range of vaccines to their communities. Pneumococcal, meningococcal, human papillomavirus, rotavirus, and a second dose of measles-containing vaccine are just some of the examples of vaccines now given to children in Ghana.

Expansion of the number of vaccines delivered has also required accompanying increases in cold chain equipment capacity, health care worker training, and community engagement to assure the public and individual parents support the program. As systems become more resilient, options for incorporating new vaccines by flexing the system’s characteristics become more likely. For example, the first licensed malaria vaccine, Mosquirix™, will likely require a regimen of up to 4 doses and with three of the 4 doses given at ages that are not currently part of the routine timing of immunization visits for well children in many highly endemic countries. Most infant vaccines in the EPI are given at ages 6, 10, and 14 weeks and age 9 months. The Mosquirix™ regimen is likely to begin at about age 5 months of age and deliver four doses in total between ages of 5 and 18 months, a period where only one typical immunization contact currently exists in many of the vaccine programs where the vaccine may be deployed. In the era of global convergence, stronger, more resilient immunization systems will be better equipped to accommodate new vaccines into their programs by flexing the contact points to accommodate the added injections or maximize the immunologic properties of the vaccine.

### 1.5 Value for money

For the past 40 years, the risk to children in developing countries from death due to vaccine preventable diseases like measles, meningitis, pneumonia or diarrhea has been substantial and far greater than the risk in wealthy countries. This combination of absolute risk rates and relative inequalities, combined with the relatively cost-effective investment in vaccines, has enabled justification of large sums of international aid to support vaccination programs in low-income or even lower-middle income countries. This simple, humanitarian crisis type of justification will be increasingly difficult to justify in the era of the grand convergence and decreasing disease risk.

As the grand convergence analysis predicts, we can expect that overall in most developing countries the trend will be toward declining child mortality rates. Furthermore, it’s likely that this trend will be most substantially observed in children aged 1–4 years old and a slower rate of decline in newborn mortality. In these environments, the emphasis on ‘life-saving’ interventions will shift toward newborn mortality, where vaccines may play a role by reducing the incidence of respiratory syncytial virus and group B streptococcal diseases but this decrease is likely to be far smaller than the historical impact of vaccines like measles on overall child mortality.

Also in the grand convergence era it may be more difficult to justify incremental investments of domestic resources in immunizations. The value for money for immunization procurement will compete with other more horizontal investments like environmental improvements in water and sanitation, or health systems strengthening to help deliver a broad package of interventions and impact a wide range of conditions rather than just one disease.

### 1.6 Community ownership and individual normative behaviors

The strongest immunization programs are often the ones where there is a strong tradition of normative behaviors that demand immunization as a core service and leadership that prioritize the delivery of services including immunizations to their communities. In an increasingly fragmented world with an increasing number of middle income countries, the role of externally driven programs should be expected to decrease. Consequently, the role of strong political will in local and national governments will be essential. Plainly put, we’ll be far less reliant on a single or few international organizations to drive global programs and more reliant on alignment of independent national decisions, and to a large extent, the behaviors of every individual in relation to their vaccine-seeking behavior.

Projecting so far into the future is inherently fraught with uncertainty. Twenty years hence, with the benefit of hindsight, the drivers listed above may or may not have been as important as they seem from today’s perspective. However, assuming that these drivers will impact the future of global vaccination, there are some actions that can be taken now to shape that future in ways that lead to a more robust global immunization system for the era of ‘grand convergence’ in global health.

## 2 Implications- what needs to be done?

### 2.1 Agile and flexible vaccine development platforms

Robust vaccine development capacities and streamlined regulation based on regulatory science will be important to assure that new vaccines can be developed with the same speed that new epidemic diseases emerge and create health and economic consequences. Epidemic diseases will continue to emerge with cycles of transmission in periods of weeks or months. Changing the cycle time for development and approval of vaccines to match the biology of the organisms is one way to assure a strong case for immunization in the future. Obviously substantial changes would be needed in the ways that vaccines are developed, tested, manufactured and regulated to achieve this alignment in cycle times but the advantages would be obvious.

### 2.2 Strengthen delivery systems

Simply put stronger delivery systems are going to be needed to deliver a wide range of vaccines against a broader range of illnesses and all along the life course of an individual. In addition, strong local capacities to deliver vaccines both through routine services and via mass administration in the face of epidemics will be important to future successful control of vaccine preventable diseases. This seems a simple statement but requires a coordinated, long-term effort to succeed. There is enough time for these efforts to support a role for immunizations in the era of grand convergence if we begin this work now.

### 2.3 Define and measure the broader value of vaccination

In anticipation of an era when deaths due to vaccine preventable diseases are uncommon, we can begin now by building a solid evidence base for the broader health effects of vaccinations that demonstrates their value for money beyond just prevention of illness or death. Measuring the effects that vaccines have on households is a good place to start. Are girls in households of vaccinated children more likely to stay in school? Does vaccination protect households against financial crises that raise their risk of falling into poverty even as they are climbing out? But it could also look at the longer-term effects on individuals. Are the babies born to fully immunized mothers larger at birth, and if so, do they have fewer illnesses subsequently? Many more examples of this broader valuation of vaccines are no doubt possible. Given that the evidence base will take time to build, it makes sense to begin the work now.

### 2.4 Make vaccines as safe as possible

Technologic innovations make it possible to increasingly explore the frontiers of vaccine safety, and in an era when vaccine-related adverse reactions are more likely to be on the news than vaccine-preventable disease cases, it makes sense to aim for the safest possible vaccines. This may require trade-offs that have not been made in the past, for instance, we may have to pay more for vaccines that are no more effective at disease prevention than current vaccines but less likely to produce adverse events that will lead people to discontinue their commitment to vaccinations.

### 2.5 Improve local institutional capacity for evidence-based policy decisions and program management

Institutional capacity building is one of the most difficult areas in all of global development. While there are few if any tried and true solutions to fostering the growth of strong indigenous institutions in developing countries, the one thing all can agree on is this: it takes years and years to do well. Beginning now with explicit intention to grow the capacity of local institutions, rather than substitution through external ones, increases the likelihood that we’ll have the robust ecosystem of national authorities needed to set smart policies and manage their programs to achieve them. The value of these investments will be apparent in many ways but especially in times of crisis like when a vaccine program experiences an adverse event controversy or an unexpected epidemic and populations look for credible, informed voices to explain the situation.

### 2.6 Increase local ownership of vaccination programs

Of all the implications this may be the most difficult one and at the same time the one that makes more difference than any other. Local ownership of vaccination programs requires that the national and local authorities prioritize immunization programs as a part of their essential package of services for their communities and that they constantly drive for continuous improvement of the program. This transition will be most difficult in the countries and communities that have become most dependent on external support so beginning now in arguably the most difficult places is going to be key if this ambition is to be successful.

This list of possible drivers and possible implications and actions is neither comprehensive nor exclusive. Others could equally add or delete from this list with legitimate bases for their point of view. However, one thing is clear: the world of global health is expected to change rapidly and somewhat predictably in the next twenty years. To achieve the full potential of vaccines in the era of global convergence in public health and in minimizing disease morbidity and mortality, our approach to global vaccination should likewise be expected to change and adapt. The list of drivers presented here and the consequent actions provide one way of anticipating the overall changes in global health and preparing for continued contributions of vaccines and immunization systems in an era of global health convergence (see Table 1).

## Conflict of interest

None declared.

## Figures and Tables

**Table 1:** Key drivers for future vaccine decision making, implications and potential barriers.

Driver	Implications	Potential barriers
Diseases with epidemic potential	Strong capacities to react to epidemics through agile vaccine development and regulation, and mass administration to achieve high coverage levels	Lack of political will and global coordination between outbreaks; regulatory hurdles create long cycle times for new vaccines that diminish their value in a crisis
Targeting local epidemiologic needs	Need to understand current needs and model likely future impact of diseases given global warming and other demographic trends	Lack of strong surveillance capacity in highly affected areas and local institutions that lack capacity to use data to drive vaccine development and use
Delivery system strength	Need to link system strengthening to new vaccine implementation	Need for political will to prioritize and sustain funding and to drive accountability for results.
Vaccine safety	Make vaccines as safe as possible and be clear about the risks and benefit associated with vaccines and disease.	Increasing anti-vaccine movements, lack of coordinated communication, need for better risk/benefit communication
Value for money	Need a broad based assessment of value that takes all benefits into account	Entrenched concepts on limited based for CE analyses, competing priorities better understood or easier to quantify
Community ownership and individual normative behaviors	For new vaccines or new systems, need to obtain community buy in prior to implementation to increase local ownership	Lack of understanding on what determines vaccine confidence, lack of coordinated strategy
